# A bioinformatics screen identifies TCF19 as an aggressiveness‐sustaining gene in prostate cancer

**DOI:** 10.1002/1878-0261.70118

**Published:** 2025-09-15

**Authors:** Amaia Ercilla, Jana R. Crespo, Saioa Garcia‐Longarte, Marta Fidalgo, Sara del Palacio, Natalia Martin‐Martin, Onintza Carlevaris, Ianire Astobiza, Sonia Fernández‐Ruiz, Marc Guiu, Laura Bárcena, Isabel Mendizabal, Ana M. Aransay, Mariona Graupera, Roger R. Gomis, Arkaitz Carracedo

**Affiliations:** ^1^ Center for Cooperative Research in Biosciences (CIC bioGUNE) Basque Research and Technology Alliance (BRTA) Derio Spain; ^2^ Centro de Investigación Biomédica En Red de Cáncer (CIBERONC) Madrid Spain; ^3^ IKERBASQUE, Basque Foundation for Science Barcelona Spain; ^4^ Endothelial Pathobiology and Microenvironment Group Josep Carreras Leukaemia Research Institute (IJC) Barcelona Spain; ^5^ Cancer Science Program, Institute for Research in Biomedicine (IRB Barcelona) The Barcelona Institute of Science and Technology Spain; ^6^ Translational Prostate Cancer Research Lab, CIC bioGUNE‐Basurto Biobizkaia Health Research Institute Spain; ^7^ CIBERehd, Insituto de Salud Carlos III Madrid Spain; ^8^ ICREA, Institució Catalana de Recerca i Estudis Avançats Barcelona Spain; ^9^ School of Medicine Universitat de Barcelona Spain; ^10^ Biochemistry and Molecular Biology Department University of the Basque Country (UPV/EHU) Bilbao Spain

**Keywords:** androgen signaling, meta‐analysis, metastatic prostate cancer, transcription factor 19, vascular permeability

## Abstract

Prostate cancer is a prevalent tumor type that, despite being highly curable, progresses to metastatic disease in a fraction of patients, thus accounting for more than 350 000 annual deaths worldwide. In turn, uncovering the molecular insights of metastatic disease is instrumental in improving the survival rate of prostate cancer patients. By means of gene expression meta‐analysis in multiple prostate cancer patient cohorts, we identified a set of genes that are differentially expressed in aggressive prostate cancer. *Transcription factor 19 (TCF19)* stood out as an unprecedented epithelial gene upregulated in metastatic disease, with prognostic potential and negatively associated with the activity of the androgen receptor. By combining computational and empirical approaches, our data revealed that TCF19 is required for full metastatic capacity, and its depletion influences core cancer‐related processes, such as tumor growth and vascular permeability, supporting the role of this gene in the dissemination of prostate tumor cells.

## Graphical abstract

Gene expression meta‐analysis in multiple prostate cancer patient cohorts identifies *Transcription factor 19 (TCF19)* as an aggressiveness‐sustaining gene with prognostic potential. *TCF19* is a gene repressed by androgen signaling that sustains core cancer‐related processes such as vascular permeability or tumor growth and metastasis.

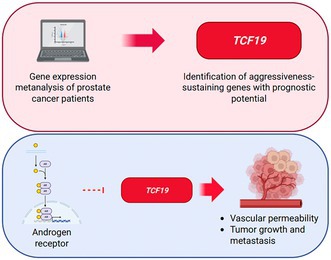

**Abbreviations**
AAALAC: Accreditation of Laboratory Animal Care
ADT: androgen deprivation therapy
AIC: Akaike Information Criterion
AR: androgen receptor
ARPI: androgen receptor pathway inhibitor
CRPC: castration‐resistant prostate cancer
DEG: differentially expressed gene
DHT: dihydrotestosterone
DMEM: Dulbecco's modified eagle medium
DSMZ: Deutsche Sammlung von Mikroorganismen und Zelkulturen GmbH
EDU: 5‐etinil‐2′‐desoxiuridina
EMT: epithelial‐to‐mesenchymal transition
ES: enrichment score
FBS: fetal bovine serum
FC: fold change
FHA: forkhead‐associated domain
GEO: Gene Expression Omnibus
GSEA: gene set enrichment analysis
HR: hazard ratio
M: metastasis
mPCa: metastatic prostate cancer
N: nontumoral
NuRD: nucleosome‐remodeling‐deacetylase
PCa: prostate cancer
PHD: plant homeodomain
PT: primary tumor
RPMI: Roswell park memorial institute
SEM: standard error of mean
shRNA: short hairpin
TCF19: transcription factor 19
αSMA: alpha smooth muscle actin

## 1 Introduction

Prostate cancer (PCa) is the second most common cancer type in males worldwide, affecting millions of men every year. Despite being highly curative, due to its high incidence, PCa accounts for more than 350 000 annual deaths, making it one of the leading causes of cancer‐associated deaths in the gender [1]. The incorporation of computational biology approaches over the last years has provided a more multidisciplinary view to PCa research, increasing our understanding of the molecular basis of this disease. For instance, pan‐cancer whole genome sequencing studies have shown that, in contrast to other tumor types, the genomic landscape of advanced disease differs profoundly from the indolent tumors [2]. Despite these advances, the 5‐year survival rate of patients that suffer the most aggressive form of metastatic PCa remains below 30–40%, and the key determinants of its aggressiveness still need to be elucidated [1].

Androgen receptor (AR) plays a central role in the differentiation and homeostasis of the prostate tissue. Although it is commonly considered a transcription activator, increasing evidence supports an additional role of AR as a transcriptional repressor [3]. AR activity is commonly hijacked by tumor cells to support growth, underscoring the relevance of targeting this receptor in prostate cancer. However, although androgen deprivation therapy initially shows a favorable response, resistance eventually arises, leading to castration‐resistant prostate cancer (CRPC) [1].

Transcription factor 19 (TCF19) was originally identified as a serum‐regulated factor expressed at the G1/S boundary and S phase [4]. It harbors a forkhead‐associated (FHA) domain and a plant homeodomain (PHD) finger through which it binds to lysine 4‐trimethylated histone 3 [5]. Genome‐wide association studies postulated a role for TCF19 in type I and II diabetes [6, 7, 8], and more recent studies have reported its relevance in regulating glucose metabolism [9, 10, 11], including the recruitment of the nucleosome‐remodeling‐deacetylase (NuRD) complex to the promoters of genes involved in *de novo* glucose production [5]. Besides controlling the recruitment of NuRD, TCF19 can form different transcription activation/repression complexes with p53 to regulate glycolysis and OXPHOS pathways [12].

During the past years, increasing evidence supports the contribution of TCF19 to tumor progression by controlling cellular proliferation in several cancer types [13, 14, 15, 16, 17, 18, 19, 20] through a mechanism that is mediated, at least in part, by targeting the FOXO1 pathway [19, 20]. Very recently, the roles of TCF19 that go beyond promoting cellular proliferation, such as inducing epithelial‐to‐mesenchymal (EMT) transition [21] or facilitating CD8 T cell exhaustion [22], have also been reported. Despite these recent advances in the role of TCF19, its contribution to prostate cancer progression, and in particular to metastatic disease, has never been addressed before.

In this study, we took advantage of publicly available PCa transcriptomics data and designed an integrative multi‐dataset meta‐analysis to uncover novel drivers of aggressive PCa. Using stringent criteria, we identified 9 genes whose expression (1) was consistently deregulated in metastatic specimens and (2) exhibited prognostic potential. *TCF19* stood out among the most robust epithelial genes fulfilling these criteria, and we proceeded to study its regulation and the consequences of its perturbation in prostate tumor cells.

## 2 Materials and methods

### 2.1 Gene expression data meta‐analysis

The meta‐analysis was performed with available information downloaded from Gene Expression Omnibus (GEO) with the exception of the TCGA dataset, which was obtained from (https://gdac.broadinstitute.org/). The preprocessed dataset normalization was reviewed and corrected when required. A background correction, Log_2_ normalization, and quartile normalization were applied when needed. In the Cox plots, the optimal degree of freedom was determined based on the Akaike information criterion (AIC) to fit smooth curves to the data for each gene and dataset. A nonparametric Spearman correlation test was applied to analyze the association between two genes or signatures. Patient data‐containing public datasets were stratified based on the mean *TCF19* levels to perform Gene Set Enrichment analysis (GSEA). The data from our RNA‐seq analysis was stratified based on shScramble or shTCF19 expression for GSEA [23] and xCell [24] analysis.

### 2.2 Cell culture and treatments

Human prostate cancer PC3 (RRID:CVCL_E2RM), DU145 (RRID:CVCL_0105) and LNCaP (RRID:CVCL_0395) cells were purchased at Leibniz Institut DSMZ (Deutsche Sammlung con Mikroorganismen und Zelkulturen GmbH), with the corresponding certificate of authenticity. Human prostate cancer cell lines 22Rv1 (RRID:CVCL_1045) and VCaP (RRID:CVCL_2235) were purchased from American Type Culture Collection, which provided an authentication certificate. Human prostate cancer cell C4‐2 (RID:CVCL_4782) cells were a kind gift provided by the laboratory of Dr. Pier Paolo Pandolfi. Cell lines were subjected to microsatellite‐based identity validation in 2019, and low‐passage validated cell lines were stored in liquid nitrogen. For the experiments in this study, validated cells were thawed and employed. Cell lines were tested for mycoplasma contamination routinely using MycoAlert detection Kit (LT07‐318; Lonza, Basel, Switzerland), and experiments were performed in mycoplasma‐negative cells. PC3, DU145, and VCaP cells were cultured in Dulbecco's modified Eagle medium (DMEM) (41 966–029; Gibco™, Grand Island, NY, USA). LNCaP, 22Rv1, and C4‐2 cells were maintained in Roswell Park Memorial Institute (RPMI 1640; 11 875–093) medium. Both culture mediums were supplemented with 10% of inactivated fetal bovine serum (FBS) (10 270–106; Gibco™) and 1% of penicillin/streptomycin (15 140–122; Gibco™). MDV3100 was purchased from Santa Cruz Biotechnology (sc‐364 354) and prepared according to the vendor instructions.

### 2.3 Lentiviral production and cell line generation

HEK293FT cells were used for lentiviral production. Lentiviral vectors expressing short hairpins (shRNAs) against human *Scramble and TCF19* were purchased from Sigma‐Aldrich (St. Louis, MO, USA). Cells were transfected with lentiviral vectors following standard procedures. Puromycin (2 μg·mL^−1^; Sigma‐Aldrich; P8833) was used for selection. The shRNA sequences are detailed in Table S1.

### 2.4 Cellular assays

#### 2.4.1 Proliferation assays

Proliferation assays were performed as previously described [25]. Briefly, 5000 cells per well were seeded in 12‐well plates in triplicate. Cells were fixed with 10% formalin (Avantor, Radnor, PA, USA) after 0, 2, and 4 days and stained with crystal violet (0.1% in 20% methanol; Sigma‐Aldrich) for 30 min. Plates were air dried for at least overnight before dissolving the crystal violet in 10% acetic acid for reading the absorbance at 595.

#### 2.4.2 Cell cycle analysis

For cell cycle analysis, cells were seeded in black‐wall 96‐well plates (BIO‐Greiner) and labeled with 10 μm 5‐etinil‐2′‐desoxiuridina (EdU, A10044; Invitrogen, Waltham, MA, USA) for 30 min before fixing them with 4% formaldehyde. For EdU detection, cells were permeabilized with 0.2% triton‐containing PBS for 30 min, and a click reaction was performed by incubating the cells for 1 h at RT on click‐IT buffer [100 mm Tris–HCl pH 8, 2 mm CuSO4 (Sigma‐Aldrich), 1 ng Alexa Fluor 488 Azide (A10266; Life Technologies), and 100 mm ascorbic acid (PHR1008‐2G; Sigma‐Aldrich)]. Mitotic cells were detected by immunostaining with Phospho‐Ser/Thr‐Pro MPM‐2 (05–368; Sigma‐Aldrich) antibody. DNA was counterstained with Hoechst (H3570; Life technologies, Carlsbad, CA, USA).

#### 2.4.3 Foci and colony formation assays

Foci and colony formation assays were performed as previously described [25].

In brief, the ability to grow individualized cells was measured by seeding 250 cells/well (DU145, PC3, 22Rv1), 5000 cells/ well (C4‐2), and 7500 cells per well (LNCaP) in a 6‐well plate in triplicate. Cells were fixed with 10% formalin (Avantor) and stained with 0.1% crystal violet (Sigma‐Aldrich) in 20% methanol after 7 (DU145, C4‐2, and LNCaP) or 15 (PC3, 22Rv1) days. The number of foci was counted using the Fiji software. For the crystal violet solubilization, 10% acetic acid was used. The intensity of each well was quantified by measuring the absorbance at 595 nm.

Anchorage‐independent growth was measured by seeding 2500 (PC3) or 5000 (DU145, 22Rv1) cells on agar‐coated 6‐well dishes in triplicate as previously described [25]. Colonies were imaged using an Olympus IX‐83 inverted microscope operated by the CellSens software, and the number of visible colonies was counted using the Fiji software.

#### 2.4.4 Wound healing assays

Cell migration rate was measured as previously described [25]. Briefly, cells were seeded at high confluency on 6‐well plates in triplicate; a wound was created with the help of a pipet tip, and images were acquired at different timepoints to calculate the linear growth of wound closure. The Fiji software was used to quantify the wounded area.

#### 2.4.5 Invasive growth assays

Spheroid assays were performed as previously described [25] by preparing 700 cell drops of 25 μL with 6% methylcellulose (M0387; Sigma‐Aldrich) and incubating them at 37 °C and 5% CO_2_ for 48 h. Spheroids were collected and embedded in collagen I solution (5005; Advanced BioMatrix PureCol, Carlsbad, CA, USA). Pictures were taken at the indicated timepoints using an Olympus IX‐83 inverted microscope operated by the CellSens software. Invasive growth was calculated by the differential area between initial and final timepoints using the FiJi software.

### 2.5 *In vivo* assays

*In vivo* assays were performed as previously described [25]. All mouse experiments were carried out following the ethical guidelines established by the Biosafety and Animal Welfare Committee at CIC bioGUNE (Spanish acronym for center for cooperative research in Biosciences). The procedures employed were carried out following the recommendations from the Association for Assessment and Accreditation of Laboratory Animal Care (AAALAC), and the study was approved by the Bioethics and Welfare committee under the code P‐CBBA‐0121. Mice were purchased from Envigo and maintained in controlled environmental conditions, with time‐controlled lighting on standard 12:12 light:dark cycles, 30–50% humidity, and controlled temperature at 22 ± 2 °C. Diet and water were provided *ad libitum*. Orthotopic xenotransplant assays were performed by injecting 7.5 × 10^5^ GFP‐Luc expressing PC3 cells into the ventral prostate lobes of 6‐week‐old Nu/Nu immunodeficient males (Hsd:AthymicNude‐Foxn1nu from Envigo). 1.5 × 10^5^ GFP‐Luc expressing PC3 cells were injected in the left ventricle of 10‐week‐old Nu/Nu immunodeficient males (Hsd:AthymicNude‐Foxn1nu from Envigo) for intracardiac metastatic assays. The sh2 was used in both cases to generate TCF19‐depleted cells. Tumor growth and dissemination were followed by measuring bioluminescence with IVIS technology (PerkinElmer, Shelton, CT, USA) for the indicated days. Intra‐orbital injections of 50 μL luciferase at 15 mg/mL were used during the follow‐up.

### 2.6 Real‐time quantitative RT‐PCR (qRT‐PCR)

Maxwell RSC simplyRNA Cells Kit (AS1390; Promega, Madison, WI, CA) was used to isolate total RNA. Complementary DNA was produced using Maxima™ H Minus cDNA Synthesis Master Mix (M1682; Invitrogen) following the manufacturer's guidelines. qRT‐PCR was run in a Viia7, QS5, or QS6 Real‐Time PCR Systems (Applied Biosystems) using the Taqman probes from Life Technologies or the primer/probes from the Universal Probe Library from Roche detailed in Table S2. The expression of individual genes was calculated and normalized to the indicated reference gene.

### 2.7 Immunohistochemistry and immunofluorescence experiments

For the analysis of Ki67, P‐pRb (S807/811) and Cleaved‐Caspase 3 levels in the tumor sections of the orthotopic experiments, paraffin‐embedded tissues were deparaffinized and rehydrated, and antigen retrieval was performed using citrate pH = 6 buffer following standard procedures. Tissue sections were permeabilized by incubation with 0.2% triton‐containing PBS for 30 min at RT and blocked with 2% FBS‐1% BSA‐containing PBS. For IHC, tissues were incubated with Ki67 (ab16667; Abcam, Cambridge, UK) antibody overnight at 4 °C and 1 : 100 dilution. Hematoxylin/eosin was applied as counterstaining. Secondary antibody and DAB staining were performed by routinely used procedures. For IF, primary antibodies against Phospho‐Ser/Thr‐Pro MPM‐2 (05–368; 1 : 250; Sigma‐Aldrich), P‐pRb S807/811–647 (#8974; 1 : 50; Cell Signaling, Danvers, MA, USA) or Cleaved‐Caspase 3 (#9661; 1 : 400; Cell Signaling) were incubated for 1 h or 2 h (in the case of Cleaved‐Caspase 3), followed by a 1‐h incubation with Alexa Fluor (1 : 500; Thermo Fisher scientific, Waltham, MA, USA) secondary antibodies. DNA was counterstained with Hoechst 1 : 2000 (H3570; Life technologies).

For the analysis of CD31, Alpha Smooth Muscle Actin (αSMA), TER119, B‐catenin, and Desmin levels in the tumor sections of the orthotopic experiments, paraffin sections (5 μm) were incubated at 60 °C for 1 h, deparaffinized, and rehydrated. Antigen retrieval was then performed with citrate buffer at 100 °C for 10 min, followed by permeabilization (0.2% Tween‐20 in PBS) at RT for 30 min. Sections were blocked for 30 min with 10% horse serum (HS), 2% BSA in PBS, and subsequently incubated with the primary antibodies (antibodies were diluted in 5% HS, 1%BSA, in PBS) overnight at 4 °C. The following primary antibodies were used in each case: CD31 (ab28364; Abcam), αSMA (C6198, Cy3 conjugated; Sigma‐Aldrich), TER119 (MAB1125; R&D systems, Minneapolis, MN, USA), B‐catenin (610 154; BD Transduction Laboratories, New York, NY, USA) and Desmin (ab195177, AF647 conjugated; Abcam). Alexa‐Fluor secondary antibodies from Invitrogen (diluted the same way as primary antibodies) were added for 2 h at RT, and slides were mounted using Immu‐Mount (9 990 402; Epredia, Portsmouth, NH, USA). In between steps, slides were washed three times with PBS for 5 min at RT, in motion. DNA was counterstained using DAPI (S33025; Invitrogen). Images were acquired using a Leica Stellaris 8 confocal microscope. All tile scans were acquired at 10× magnification, and images for quantification were acquired at 20× or 40× magnification. Analysis and quantification of the images were done with the Imaris software (version 10.1.1).

### 2.8 RNA‐seq analysis

Total RNA was extracted using Maxwell RSC simplyRNA Cells Kit (AS1390; Promega), and total RNA libraries were prepared at the Genomics platform of CIC bioGUNE using TruSeq Stranded Total RNA with Ribo‐Zero Globin kit (Cat.# 20 020 612; Illumina Inc.) and TruSeq RNA CD Index Plate (Cat.# 20 019 792; Illumina Inc.) following ‘TruSeq Stranded Total RNA Sample Prep‐guide (Part # 15031048 Rev. E)’. Libraries were sequenced on an Illumina NovaSeq 6000 instrument to generate at least 40 million paired‐end 100‐bp reads.

Reads were aligned to the human reference genome (hg38) using STAR (version_2.7.5c) in two‐pass mode following STAR best practices and recommendations [26]. The quality of the data was evaluated using STAR (version 2.7.5c) (26) and samtools (version 1.15) [27]. PCR duplicates were removed from aligned bam files using samtools (version 1.15) [27]. Read counts were extracted from the aligned bam files using subread's FeatureCounts (version 2.0.3) [28]. Normalization of read counts for analysis was done using the Ratio of the Variance method, which accounts for inter‐sample variance, and the differential expression analysis of the normalized read counts between the sample groups was performed following best practices and recommendations of EdgeR [29, 30] and Limma [31] on R environment (version 3.6.0). All the codes used for data analysis are available upon request.

### 2.9 Statistical analysis

Statistical analyses were performed alike previously described [32]. Briefly, no statistical method was used to predetermine sample size. The experiments were not randomized. The investigators were not blinded to allocation during experiments and outcome assessment. None of the samples/animals were excluded from the analysis. Data analyzed by parametric tests are represented by the mean ± standard error of mean (S.E.M.) of pooled experiments unless otherwise stated. Values of *n* represent the number of independent experiments performed or the number of individual mice or patient specimens. For each independent *in vitro* experiment, at least three technical replicates were used, and a minimum number of three experiments were performed to ensure adequate statistical power. In the *in vitro* experiments, normal distribution was assumed, and one‐sample *t*‐test was applied for one‐component comparisons with control and Student's *t*‐test for two‐component comparisons. For *in vivo* experiments, a nonparametric Mann–Whitney *U*‐test was used. Two‐tailed statistical analysis was applied for experimental design without predicted result, and one‐tailed for validation or hypothesis‐driven experiments. The confidence level used for all the statistical analyses was 0.95 (alpha value = 0.05). *P*‐value: *P < 0.05, **P < 0.01, ***P < 0.001.

## 3 Results

### 3.1 Identification of metastatic prostate cancer‐associated genes with prognostic potential

**Fig. 1:**
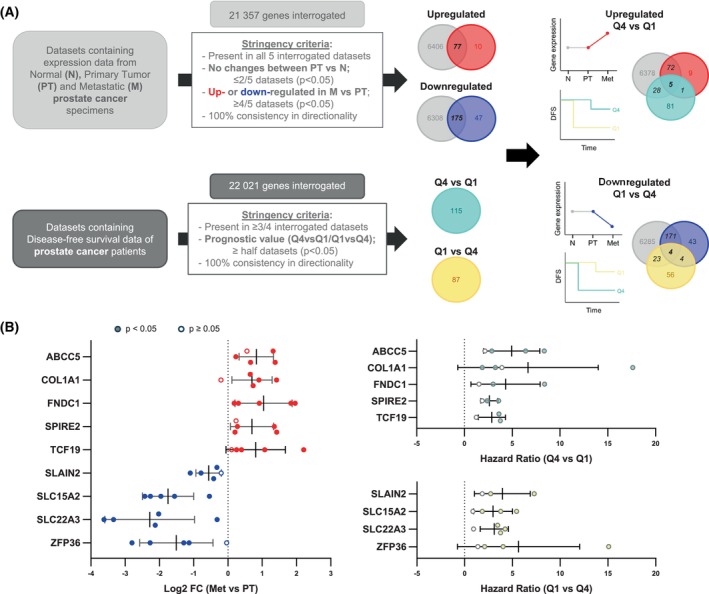
Identification of metastatic prostate cancer‐associated genes with prognostic potential. (A) Workflow of the computational screening. The stringency criteria are indicated. The Log_2_‐normalized gene expression was used to calculate the limma differential expression and select the up‐ or downregulated genes. The prognostic value of the genes was analyzed by measuring the hazard ratio (HR) between the Q1 and Q4 groups. Quartiles represent ranges of expression that divide the set of values into quarters, being Q1 and Q4 the ones with higher and lower expression, respectively. A Cox proportional hazards regression model was performed to compare the two groups. (B) The Log_2_ fold change (FC) and HR values of the candidate genes in each dataset are represented. The statistically significant results are represented with filled‐colored dots. Error bars represent SEM. *P*‐value derives from the limma differential expression between the indicated groups in the computational analysis.

To unveil novel drivers of aggressive PCa with prognostic potential, we undertook an unbiased bioinformatics strategy taking advantage of public transcriptomics datasets that contain tumor progression and patient outcome information (see workflow in Fig. 1A and cohort information in Fig. S1). We evaluated prognostic potential (the biochemical risk association—risk of recurrence of patients stratified according to the quartiles 1 vs. 4 of expression for each of the 22 021 genes present in four datasets that contained patient follow‐up information) (Fig. 1A and Fig. S1) and selected genes that showed a consistent risk association between the interrogated datasets (202 genes). To prioritize genes that were associated with disease progression and not broadly with pathogenesis, we selected genes that were altered in the course of metastasis (comparing primary tumors and metastases) but not during initiation (excluding genes consistently altered in primary tumors vs. normal tissue). We applied stringent criteria to identify the candidates that showed a more robust trend between the interrogated five datasets. From the initial 21 357 genes, we shortlisted 77 upregulated and 175 downregulated genes. When integrating differential expression in metastasis and prognostic potential, five upregulated and four downregulated genes complied with all the above criteria (individual data for these genes is presented in Fig. 1B).

### 3.2 *TCF19* is a prognostic gene upregulated in metastatic prostate cancer patients

**Fig. 2:**
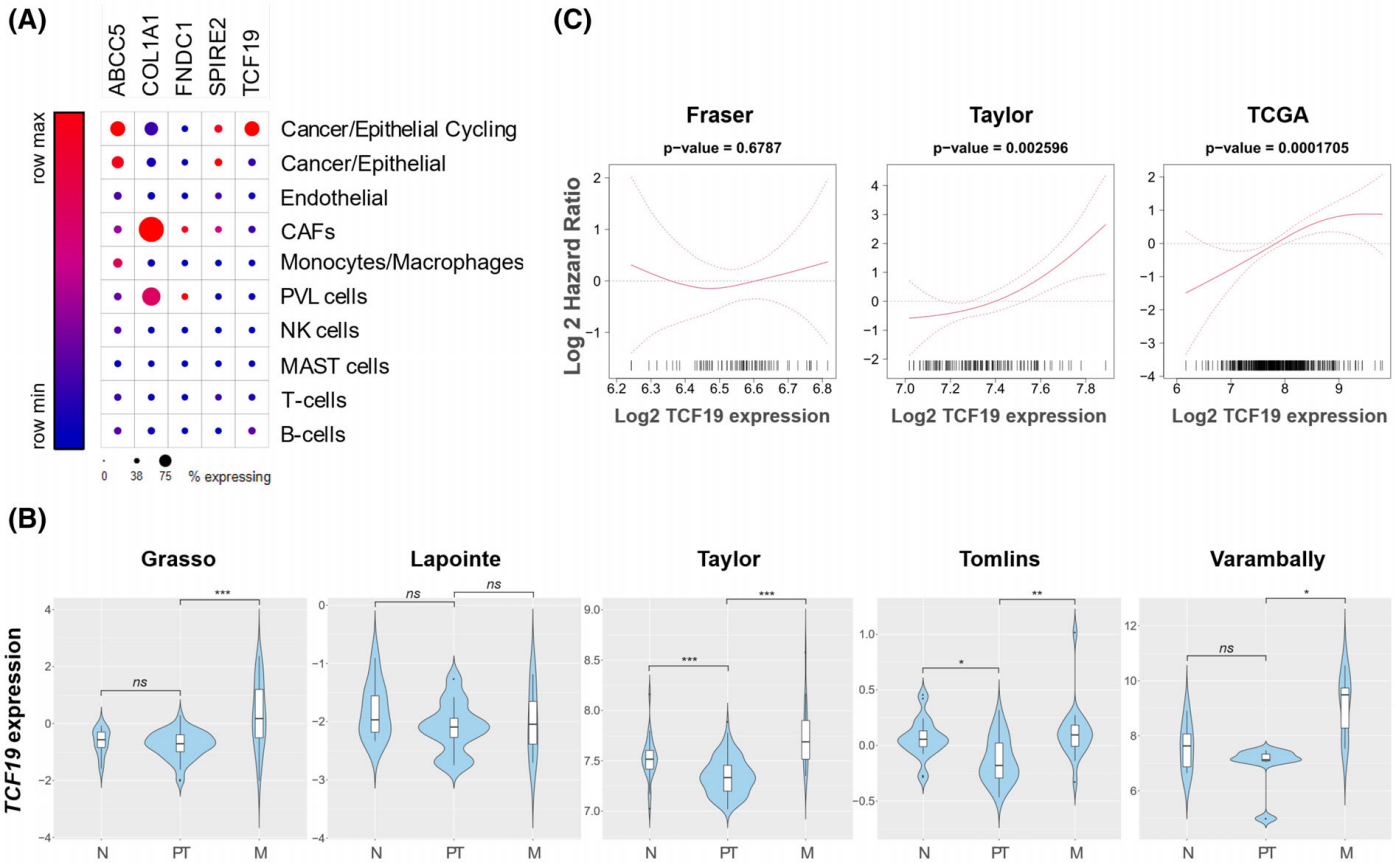
*TCF19* is a prognostic gene upregulated in metastatic prostate cancer patients. (A) The relative expression of each gene in the indicated cell type was retrieved from the single‐cell data from a prostate cancer study (PMID: 33971952). The size of the dot represents the % of expressing cells. (B) Violin plots depicting the expression of *TCF19* among nontumoral (N), primary tumor (PT), and metastatic (M) prostate cancer specimens in the indicated datasets. The *y*‐axis represents the Log_2_‐normalized gene expression (fluorescence intensity values for microarray data or, sequencing reads values obtained after gene quantification with RSEM and normalization using upper quartile in case of RNA‐seq). *P*‐value derives from the limma differential expression between the indicated groups in the computational analysis (*P*, *P*‐value. ns*P* ≥ 0.05, *P < 0.05, **P < 0.005, ***P < 0.0005). (C) Smooth hazard ratio curves. X‐axis represents the gene expression level and y‐axis represents the Log Hazard ratio. The *P*‐value indicates the significance of the association between the gene and the outcome calculated via a likelihood ratio test.

To select the gene best suited for molecular and biological studies, we focused on tumor cell‐intrinsic aggressiveness promoter genes (upregulated). We took advantage of the single‐cell portal to interrogate a single‐cell RNA‐seq dataset from PCa patients and identify the genes expressed in the epithelial compartment [33]. Of the initial nine genes, only *ABCC5* and *TCF19* met this criterion (Fig. 2A and Fig. S2). Of note, ABCC5 had been previously reported to contribute to PCa progression and androgen therapy resistance [34], validating the potential of our screening strategy to uncover aggressive PCa‐associated genes (Figs S3 and S4). In contrast, the contribution of *TCF19* to PCa progression (which had a robust and consistent association to biochemical recurrence and metastasis, Fig. 2B, C) had never been addressed before, which prompted its study.

### 3.3 *TCF19* is negatively regulated by androgen signaling in prostate cancer

**Fig. 3:**
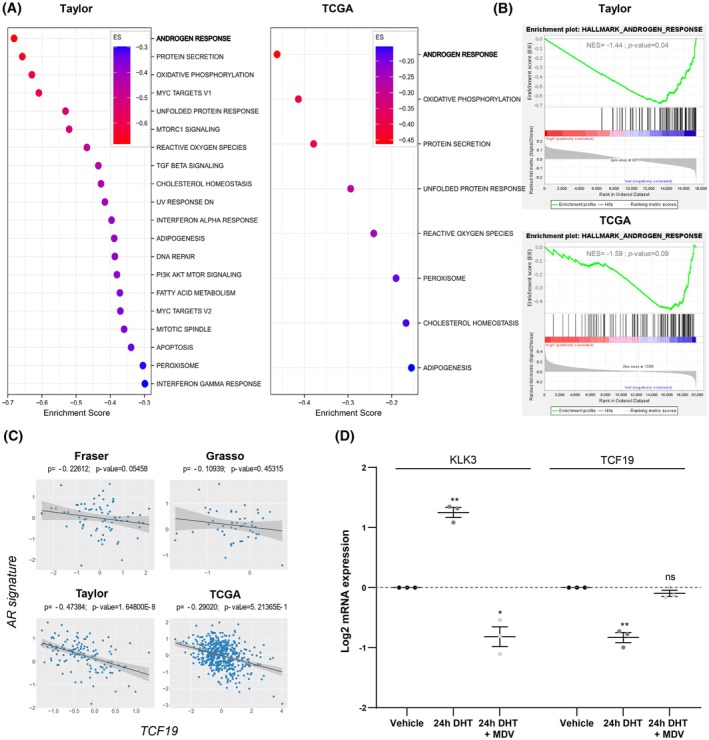
*TCF19* and androgen receptor target genes are inversely co‐expressed in prostate cancer. (A and B) GSEA of TCF19 high vs. TCF19 low patients from Taylor and TCGA datasets. Primary tumor cohorts were divided into two groups based on their mean *TCF19* mRNA levels. The pathways associated with the levels of TCF19 and the enrichment score (ES) plots of the most inversely correlated ones in each dataset are indicated. Cohort size is indicated in Fig. S5A. (C) Plotted values correspond to the Log_2_‐normalized gene expression values of *TCF19* and the AR signatures from (PMID: 34503116) (in X‐ and Y‐axis) in the primary tumor specimens from each patient in the indicated dataset. The black line represents linear regression; the gray area indicates the limits of the confidence intervals; and *P* and *P‐*value indicate Spearman's correlation coefficient and statistical significance, respectively. (D) Analysis of *KLK3* and *TCF19* expression by qRT‐PCR in the AR‐dependent LNCaP cells treated for 24 h with AR agonist (dihydrotestosterone, DHT, 10 nm) alone or in combination with the AR antagonist (enzalutamide, MDV 3100, 10 μm). Data were normalized to *GAPDH* expression and untreated (vehicle) condition. The dotted line represents the normalized value of the vehicle data. A one‐sample *t‐*test was performed. Error bars represent SEM. *n* = 3 independent experiments. *P*, *P*‐value. ns *P* ≥ 0.05, *P < 0.05, **P < 0.01.

*TCF19* is a growth‐regulated gene that has been increasingly associated with the proliferation potential of multiple cancer types [13, 14, 15, 16, 17, 18, 19, 20]. Interestingly, our screening pointed to an unprecedented role for TCF19 in PCa dissemination. To identify pathways associated with the upregulation of TCF19 in PCa, we performed GSEA [23]. We stratified the patients from two of the datasets that contained the higher number of individuals (Taylor (*n* = 131) and TCGA (*n* = 497)) into high or low TCF19 based on the mean *TCF19* expression (Fig. S5A). The GSEA analysis identified androgen response as the top repressed pathway in patients with elevated TCF19 mRNA expression (Fig. 3A, B). Of note, oxidative phosphorylation was also among the top pathways consistent with previous reports [12] and validating the strength of our approach. To confirm an inverse relationship between *TCF19* expression and AR signaling, we performed a correlation analysis between *TCF19* and an AR signature that is based on 31 genes previously developed by our group [35]. Of note, *TCF19* and the AR signature showed a robust inverse correlation in Taylor and TCGA, and a similar trend in an additional 2 datasets that contained a lower number of patients (Fig. 3C). Moreover, similar results were obtained by analyzing the correlation of *TCF19* with the AR target *KLK3* (Fig. S5B) and an additional public AR signature (Fig. S5C) [36].

AR is commonly considered a transcriptional activator. Yet, increasing evidence supports the role of AR as a transcriptional repressor [3]. To assess whether *TCF19* could be repressed by AR, we treated AR‐proficient human prostate cancer cell lines with the AR agonist dihydrotestosterone (DHT) [37]. Interestingly, DHT‐induced AR activation (Fig. 3D) resulted in the downregulation of *TCF19* in multiple PCa cell lines (Fig. 3D and Fig. S5D,E), suggesting that, directly or indirectly, AR represses *TCF19*. Of note, this effect was reverted by the addition of the AR antagonist Enzalutamide (MDV 3100) (Fig. 3D and Fig. S5E), further supporting the role of AR on *TCF19* downregulation. These results support the notion that primary tumors with lower androgen activity or those that evade the action of androgen deprivation therapy (ADT) and androgen receptor pathway inhibitors (ARPI)s through reduction in AR dependence would exhibit higher TCF19 expression levels.

### 3.4 *TCF19* silencing in prostate cancer cells reduces cell growth under stress

**Fig. 4:**
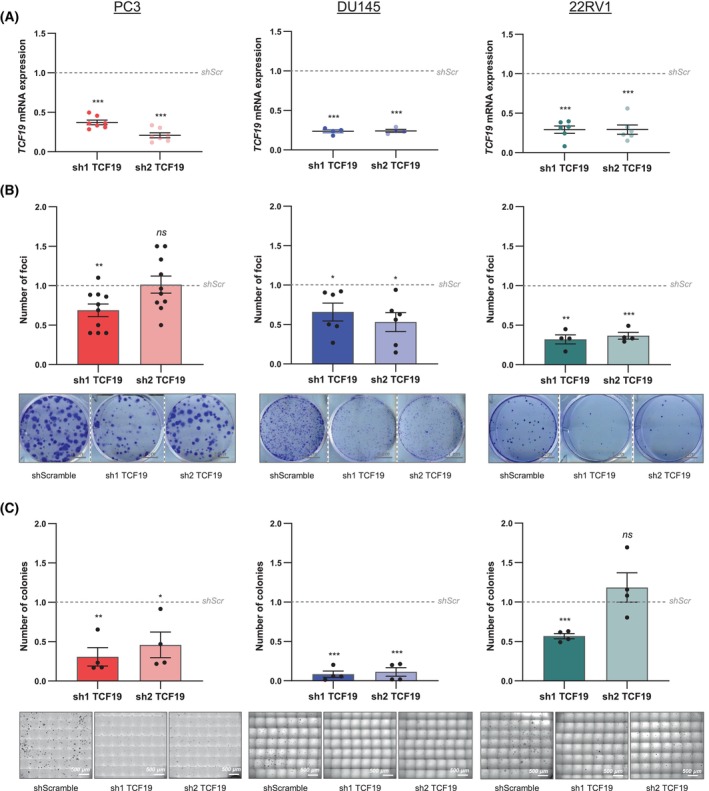
Context‐dependent growth inhibition upon *TCF19* silencing in prostate cancer cells. (A) Analysis of *TCF19* gene expression by qRT‐PCR in the indicated cell line upon silencing of *TCF19* by shRNA transduction. Data were normalized to *GAPDH* expression and shScramble (shScr) condition. The dotted line represents the normalized value of the shScramble data. A one‐sample *t‐*test was performed for statistical analysis. Error bars represent SEM. PC3 (*n* = 7), DU145 (*n* = 4), and 22Rv1 (*n* = 6) independent experiments. (B) Analysis of foci formation upon *TCF19* depletion. The number of foci normalized to shScramble condition is shown (top panels). A one‐sample *t*‐test was performed for statistical analysis. Error bars represent SEM. PC3 (*n* = 10), DU145 (*n* = 6), and 22Rv1 (*n* = 4) independent experiments. Representative images are shown (bottom panels). Scale bar: 1 cm. (C) Analysis of anchorage‐independent growth. The number of colonies normalized to shScramble is shown (top panels). A one‐sample *t*‐test was performed for statistical analysis. Error bars represent SEM. *n* = 4 independent experiments. Representative images are shown (bottom panels). Scale bar: 500 μm. *P*, *P*‐value. ns*P* ≥ 0.05, *P < 0.05, **P < 0.01, ***P < 0.001.

TCF19 depletion compromises the proliferation and foci formation capacity of colorectal [13], head and neck squamous carcinoma [38] and breast cancer [21] cell lines. To assess whether *TCF19* silencing compromises also the proliferation potential of prostate cancer cell lines, we generated stable shRNA expressing PC3, DU145, LnCaP, C4‐2, and 22Rv1 cells. The two different shRNAs used for these assays showed a similar silencing efficacy (Fig. 4A). Surprisingly, TCF19 silencing had a negligible effect on two‐dimensional cell growth and cell cycle progression under unrestricted conditions (Fig. S6A,B). Alterations in the migration and invasion ability of TCF19‐depleted cells have also been reported [13, 21, 38]. However, we did not observe consistent defects in the migration and invasion ability of human PC3 prostate cancer cells (Fig. S6C,D).

Interestingly, TCF19‐silenced PC3, DU145, 22Rv1, LnCaP, and C4‐2 cells exhibited a consistent reduction in clonal growth (colony formation assay) (Fig. 4B, Fig. S7). Moreover, TCF19‐depleted cells also showed a decreased ability to grow in anchorage‐independent conditions (soft agar colony formation assays; Fig. 4C).

### 3.5 TCF19 depletion compromises tumor growth and metastatic capacity of prostate cancer cells

**Fig. 5:**
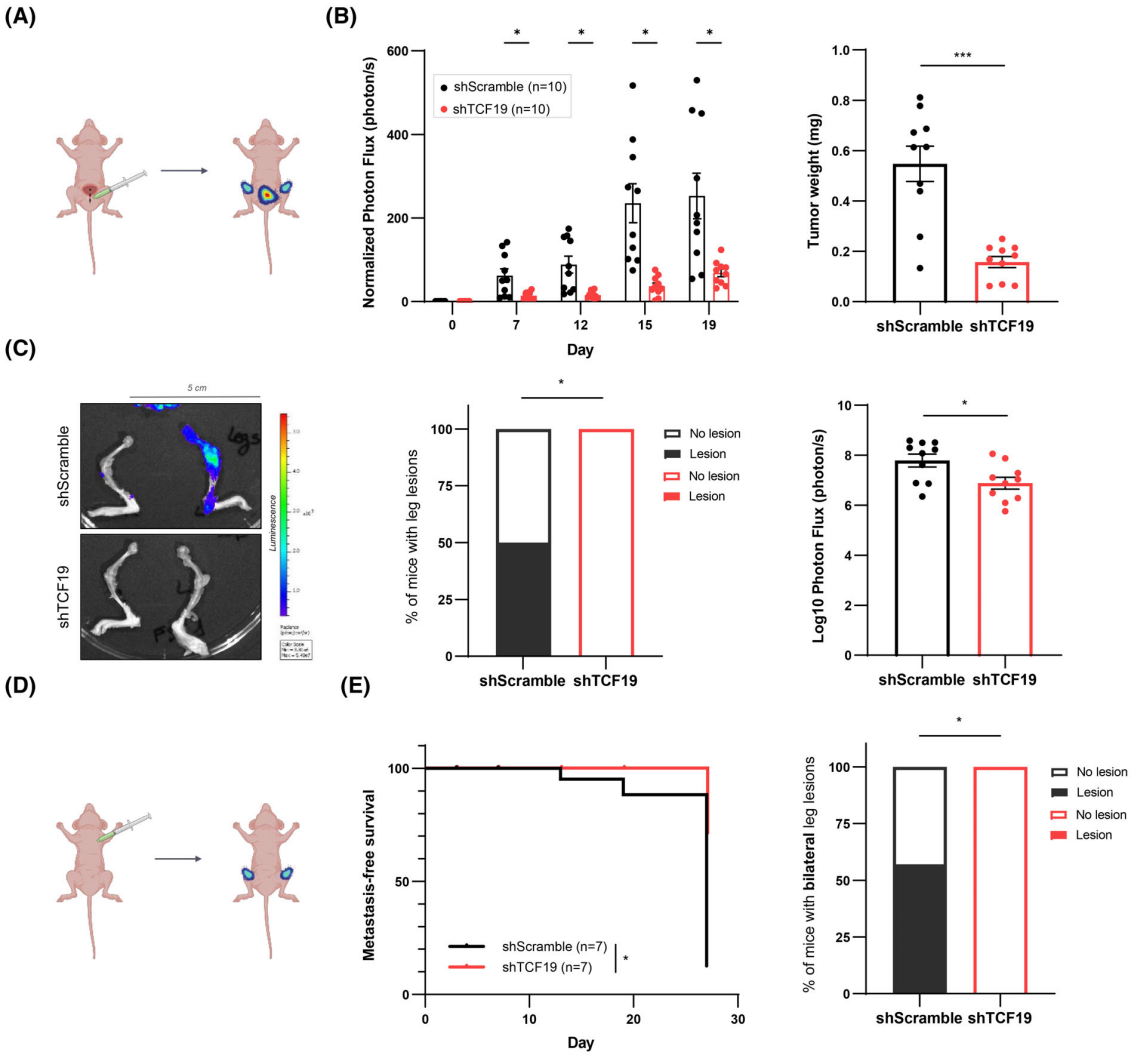
TCF19 depletion compromises tumor growth and metastatic capacity of prostate cancer cells. (A) Schematic representation of the orthotopic xenotransplant assay performed to assess the local tumor growth and the metastatic capacity of *TCF19*‐depleted PC3 GFP‐Luc cells (*n* = 10 mice per group). (B) Evaluation of local tumor growth by orthotopic xenotransplant assay. IVIS relative flux data along the experimental process (left panel). The total photon flux normalized to time 0 are represented. A multiple Mann–Whitney *U*‐test was applied for statistical analysis. *Ex vivo* tumor weight of the ventral lobes of the prostates is represented (right panel). A one‐tailed unpaired Student's *t*‐test was applied for statistical analysis. Error bars represent SEM. *n* = 10 mice per group. (C) Evaluation of metastatic lesions in the legs by orthotopic xenotransplant assay. Representative images (left panel). The *ex vivo* incidence of leg lesions is represented (middle panel). Luciferase signal above Day 0 was considered metastasis‐positive. The Log_10_ photon flux of the sum signals from both legs is represented (right panel). A two‐sided Fisher's exact test was performed. Error bars represent SEM. Scale bar: 5 cm. *n* = 10 mice/group. (D) Schematic representation of the intracardiac xenotransplant assay performed to assess the metastatic capacity of *TCF19*‐depleted PC3 GFP‐Luc cells (*n* = 7 mice per group). (E) Metastasis‐free survival curves of prostate cancer cell signal in legs were monitored for up to 27 days (left panel). Luciferase signal above Day 0 was considered metastasis‐positive. The incidence of bilateral leg lesions at Day 27 is represented (right panel). A log‐rank test and a one‐sided Fisher's exact test were performed, respectively. *n* = 7 mice/group. *P*, *P*‐value. *P < 0.05, ***P < 0.001.

In contrast to previous reports, the effect of *TCF19* silencing in human prostate cancer cell lines did not show a profound impact on regular growth and cell cycle (Fig. 4 and Fig. S6). This fact, together with the association of *TCF19* to PCa dissemination emerging from our bioinformatics screening, led us to hypothesize that the role of TCF19 in PCa might go beyond promoting cellular proliferation. Recent reports have highlighted tumorigenic roles of TCF19 in pathways different from cellular proliferation [21, 22]. To address whether this was also the case in the context of PCa, we performed two complementary *in vivo* studies. On the one hand, we performed an *in vivo* orthotopic assay (Fig. 5A). To this end, we generated stable shRNA expressing GFP‐Luc PC3 cells and injected them into the ventral lobe of the prostates of immune‐deficient nude mice. Local tumor growth and metastatic outgrowth were monitored following the luciferase‐expressing cells by IVIS. Remarkably, TCF19‐depleted cells showed a strong defect in tumor growth that correlated with a reduced primary tumor size and weight at the experimental endpoint (Fig. 5B and Fig. S8A). Moreover, the analysis of luciferase signal in distal organs *ex vivo* confirmed a lower metastatic burden in the bones of mice injected with *TCF19*‐silenced PCa cells (Fig. 5C and Fig. S8B). On the other hand, we performed a complementary *in vivo* metastatic assay. For this second approach, we transduced again GFP‐Luc expressing PC3 cells with TCF19‐targeting or Scramble shRNA and injected them intracardially in the left ventricle of immune‐deficient nude mice and monitored the luciferase signal over 27 days (Fig. 5D). Remarkably, *TCF19*‐silenced cells showed a lower number of bone lesions (Fig. 5E), confirming a role of TCF19 in sustaining metastatic dissemination in PCa.

### 3.6 *TCF19* silencing in prostate tumors results in a decrease in hypoxia‐responsive genes

To address the molecular basis of the activity of TCF19 in PCa, we first focused on the reported association with FOXO1 regulation [19, 20]. To assess whether alterations in the FOXO transcriptional program were present in TCF19‐depleted tumors (Fig. 5), we analyzed the mRNA levels of representative targets of this transcription factor in specimens collected from the *in vivo* orthotopic assay. *p27* and *CCND1* mRNA levels showed a nonsignificant trend toward elevation, and only *p21* gene expression was significantly elevated in TCF19‐silenced tumors (Fig. S9A). TCF19 depletion did not cause a significant change in the proliferation markers Ki67 or pRb^pS807/811^ *in vivo* analyzed by immunofluorescence (Fig. S9B), overall suggesting that a potential regulation of these FOXO targets did not influence the cell cycle and proliferation. The analysis of *VIMENTIN* and *SNAIL* mRNA levels in those tumors also ruled out major alterations in EMT (Fig. S9C) that contribute to metastasis in breast cancer [21]. We did not observe an increase in the apoptosis marker Cleaved‐Caspase 3 either in TCF19‐depleted tumors (Fig. S9D).

**Fig. 6:**
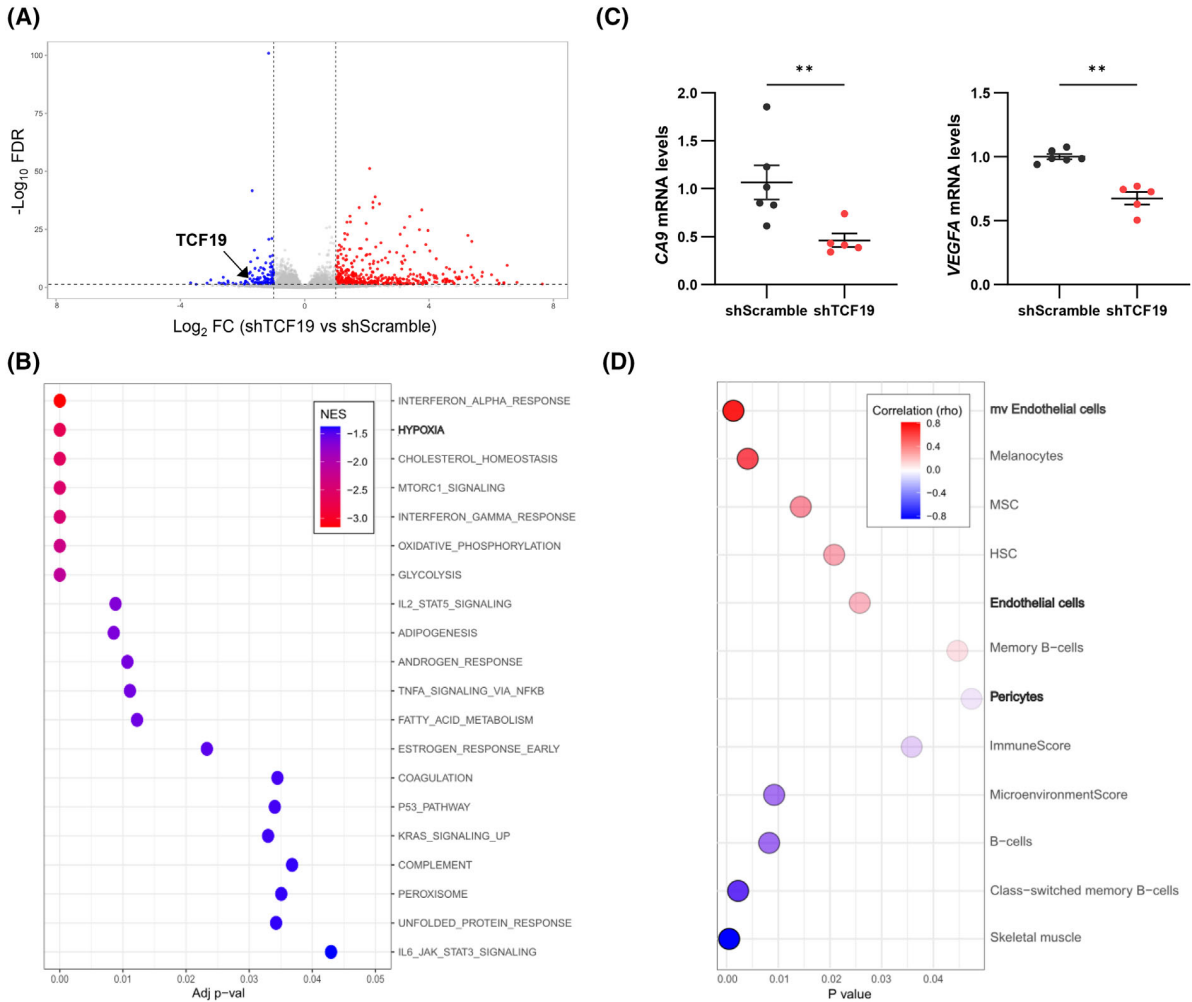
*TCF19* silencing in prostate tumors results in a decrease in hypoxia‐responsive genes. (A) Volcano plot representation of the differentially expressed genes (FDR <0.05; Log_2_FC > I1I) in the tumors collected from the orthotopic xenotransplant assay. The dot representing *TCF19* expression is highlighted. (B) A GSEA analysis was performed to identify pathways enriched upon *TCF19* depletion. The top 20 pathways phenotypically enriched in the control at the GSEA analysis are shown. (C) Gene expression analysis of shScramble and shTCF19 tumors from the *in vivo* orthotopic experiment. The tumors selected for the qRT‐PCR analysis are indicated in Fig. S7. Data were normalized to *RPLP0* expression and shScramble condition. A Mann–Whitney *U*‐test was performed for statistical analysis; error bars represent SEM. (D) Digital dissection of the tumor microenvironment in the *in vivo* orthotopic tumors from Fig. 5 using xCell (PMID: 29141660). The stromal compartments positively and negatively correlated with TCF19 were inferred from the RNA‐seq data. *P*, *P*‐value. **P < 0.01.

The analysis of previously reported processes did not shed light on the role of TCF19 in sustaining PCa metastasis. Hence, we decided to perform a high‐throughput transcriptomics analysis from our tumor specimens, taking advantage of the primary tumor samples from the orthotopic assay. The heatmap of the top 200 differentially expressed genes (DEG) nicely grouped the tumors from each experimental condition (Fig. S10A). By applying an FDR < 0.05 and a Log_2_FC|1| as cutoff, we identified 599 DEGs (GSE287409, Table S3). Of note, *TCF19* appeared downregulated in our analysis, which served as quality control (Fig. 6A). To identify the main pathways altered upon depletion of TCF19, we performed a GSEA analysis. Our GSEA analysis confirmed alterations in known TCF19‐related molecular programs such as interferon signaling [22], OXPHOS, and glycolysis [12] (Fig. 6B and Fig. S10B), but also identified novel potential pathways that could explain the defects observed in metastasis. Hypoxia‐responsive gene signatures are associated with biochemical recurrence [39] and hypoxia promotes the acquisition of castration‐resistant features in the context of PCa [40]. Interestingly, our GSEA analysis unveiled hypoxia as one of the main pathways affected by TCF19 depletion (Fig. 6B and Fig. S10C). We confirmed this data through the analysis of hypoxia target gene expression (*CA9* and *VEGFA*) in TCF19‐depleted tumors (Fig. 6C). A similar trend was observed in three additional hypoxia target genes (*ANGPTL4, BNIP3, SLC2A1*; Fig. S10D). Moreover, the computational dissection of the tumor microenvironment in the *in vivo* orthotopic tumors using xCell [24] uncovered endothelial cells as the stromal components exhibiting the most robust direct association with TCF19 perturbation (Fig. 6D).

### 3.7 *TCF19* ‐silenced tumors show vessel normalization with reduced vascular permeability

**Fig. 7:**
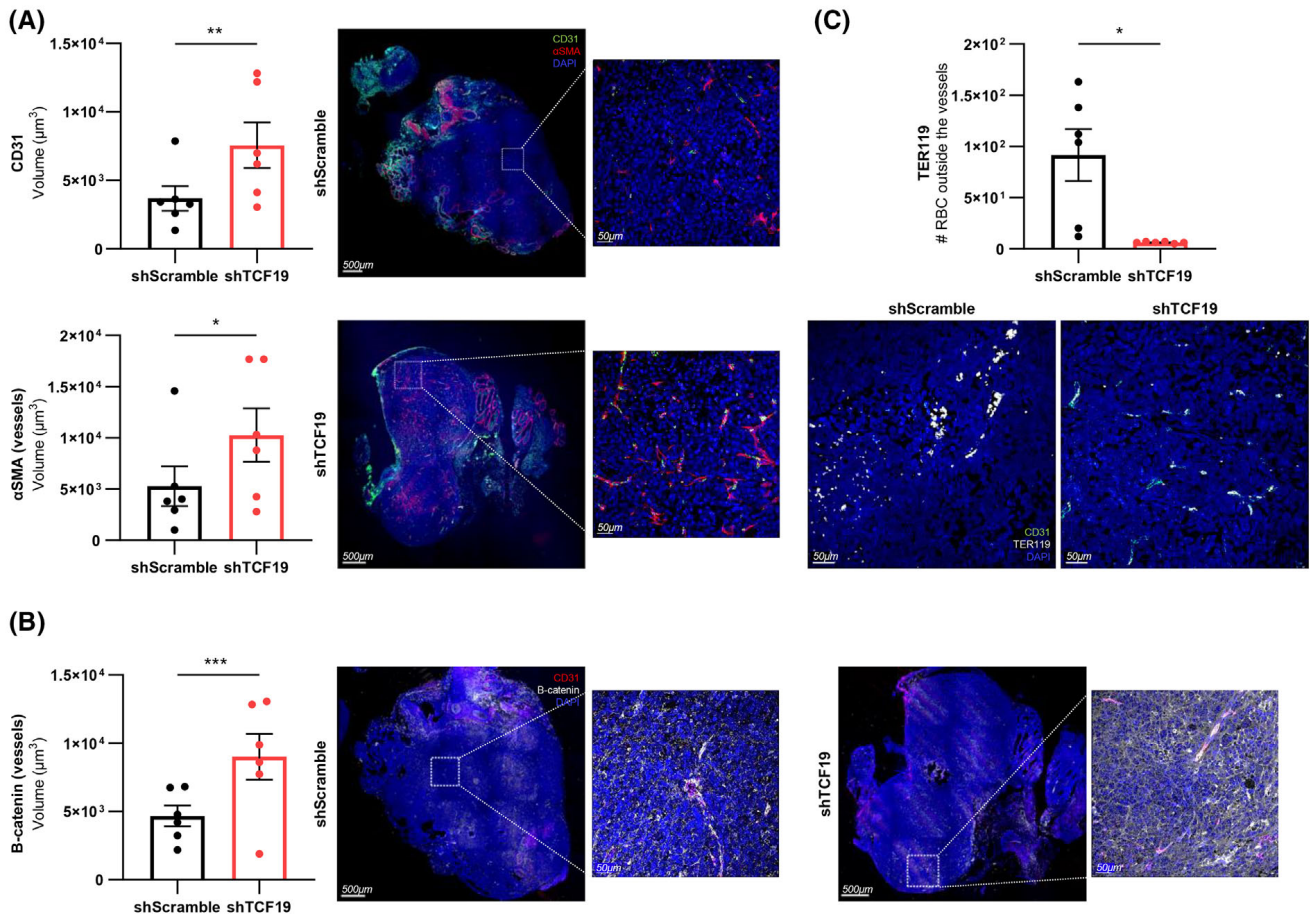
TCF19‐silenced tumors show vessel normalization with reduced vascular permeability. (A–C) Analysis of the indicated proteins by immunofluorescence in shScramble and shTCF19 tumors from the *in vivo* orthotopic experiment. CD31 and αSMA (A). B‐catenin (B). TER119 (C). The tumors selected for the IHC/IF analysis are indicated in Fig. S7 (*n* = 6 tumors per group). A nested *t*‐test was performed for statistical analysis (left panels). Error bars represent SEM. Scale bar: 500 μm and 50 μm (A and B), and 50 μm (C). Representative images are shown (right panels). *P*, *P*‐value. *P < 0.05, **P < 0.01, ***P < 0.001.

To explore whether the hypoxia and endothelial cell processes identified in molecular analyses translated to vascular alterations, we analyzed the tumors collected from the orthotopic xenotransplant assay (Fig. S8) by immunofluorescence. Endothelial abundance in the tumor area was estimated by means of CD31 staining. Counterintuitively, TCF19‐depleted tumors showed an increased number of blood vessels compared with control ones (Fig. 7A and Fig. S11). However, they exhibited distinctive structural and functional features indicative of increased functionality. We monitored the pericyte compartment in vessels to address their maturity. Interestingly, TCF19‐depleted tumors showed an increased level of αSMA around the vessels, suggestive of increased functional pericyte coverage and a potential reduction in vascular permeability that enables tumor cell intravasation (Fig. 7A) [41]. In turn, we analyzed vessel permeability indicators. On the one hand, we monitored endothelial junction tightening using the marker β‐catenin, which was elevated, suggesting tighter endothelial cell junctions (Fig. 7B). On the other hand, we analyzed erythrocyte extravasation in tumoral areas, which would be indicative of elevated vascular permeability, and found a profound reduction in extravascular Ter119 staining (Fig. 7C). Altogether, the characterization of the vascular bed in tumors revealed a regulation of vascular permeability and function upon TCF19 depletion, which is consistent with the reduced growth and dissemination of PCa.

## 4 Discussion

Recent advances in PCa research have increased our understanding of this complex disease that still causes more than 350.000 annual deaths, which are mainly due to the acquisition of androgen resistant and chemorefractory features [1]. Uncovering the key determinants of PCa aggressiveness is essential to understand the high morbidity due to metastatic disease. By analyzing patient data from public repositories, we have identified nine genes with prognostic potential that are dysregulated in metastatic PCa patients. The genes identified in our screening are expressed in epithelial and stromal cells, suggesting that both tumor cell‐intrinsic and extrinsic alterations can be indicative and/or contribute to the gain of aggressive and metastatic features.

*TCF19* ranked as a top upregulated epithelial gene in our screening. The contribution of TCF19 to tumor progression by controlling cellular proliferation has been reported in gastric cancer [14], hepatocellular carcinoma [15, 19], non‐small cell lung cancer [17, 20], colorectal cancer [13], and liver cancer [18]. In addition, TCF19 levels have been associated with the risk of head and neck cancer (38) and it has proven prognostic potential for colorectal cancer [13], renal clear cell carcinoma [42], endometrial cancer [22] and hepatocellular carcinoma [43] patients. Consistent with this notion, our patient data show a tumor and metastasis supportive role for TCF19 in PCa. Interestingly, the combination of computational, *in vitro*, and *in vivo* experimental strategies revealed that, beyond the role of TCF19 in supporting tumor‐intrinsic properties, it also contributes to tumor microenvironment remodeling. Although the exact molecular mechanism driving this phenomenon remains to be elucidated, our work provides evidence for the role of tumor cell‐expressed *TCF19* in vessel homeostasis and permeability, a process that is critical in cancer cell dissemination [44].

Inhibition of androgen signaling or AR activity elicits a transient clinical benefit, that is bypassed in patients leading to CRPC. Importantly, the mechanisms that enable prostate cancer cell adaptation to lack of AR signaling are poorly understood but could include transcriptional rewiring and cellular plasticity [1]. Indeed, a gene that ranked as a top prognostic factor in PCa progression in our screening, ABCC5, is reportedly associated with the acquisition of CRPC features [34, 38]. Our results demonstrate that AR signaling opposes *TCF19* expression. In turn, it is tempting to speculate that the upregulation of TCF19 upon treatment with ADT or AR inhibitors could represent a first adaptive response that would support prostate cancer cell function and the eventual acquisition of aggressive features. In line with our findings of TCF19‐regulated hypoxia response and vessel function, the reported contribution of hypoxia to biochemical recurrence [39] and to the acquisition of castration‐resistant features [40] could be indicative of an important role for the TCF19‐hypoxia‐vessel homeostasis in PCa progression. Future work will clarify the real contribution of TCF19 to the gain of castration‐resistant features.

## 5 Conclusions

In this study, we combine computational and empiric approaches to identify the causal role and prognostic potential of *TCF19* in aggressive prostate cancer. We find that this gene is repressed by androgen signaling and contributes to stroma remodeling by means of vessel homeostasis and permeability, a central process in cancer cell dissemination.

## Conflict of interest

The authors declare no conflict of interest.

## Author contributions

AE designed and performed the majority of experiments, data analysis, prepared the figures, and drafted the manuscript. JR‐C, MF, SdP, and S‐FR provided technical support with the *in vitro* experiments. SG‐L and IM provided bioinformatics support. NM‐M contributed to the design and data analysis of computational screening. OC, IA, and MGu provided technical support with the *in vivo* experiments. The RNA‐seq analysis was performed at the Genome Analysis Platform at CIC bioGUNE with the support of LB and AM‐A. MGr and RR‐G contributed to experimental design and supervision (MGr supervised the work of MF; RRG supervised the work of MGu) and provided valuable input. AC conceived the study, supervised the execution of the project, and drafted the manuscript. All authors have read and approved the final version of the manuscript.

## Supporting information


**Fig. S1.** Related to Fig. 1.
**Fig. S2.** Related to Fig. 2.
**Fig. S3.** Related to Fig. 2.
**Fig. S4.** Related to Fig. 2.
**Fig. S5.** Related to Fig. 3.
**Fig. S6.** Related to Fig. 4.
**Fig. S7.** Related to Fig. 4.
**Fig. S8.** Related to Fig. 5.
**Fig. S9.** Related to Fig. 6.
**Fig. S10.** Related to Fig. 6.
**Fig. S11.** Related to Fig. 7.

**Table S1.** Short hairpin RNA sequences employed in the study and Sigma Mission reference.

**Table S2.** Taqman probes (Life Technologies) and primer/probes from Universal Probe Library (Roche) used in the study.

**Table S3.** Differentially expressed genes in PC3 cells upon transduction with shTCF19.

## Data Availability

The authors declare that data supporting the findings of this study are available within the paper and its Supporting Information. RNA‐seq data are accessible in the GEO repository with ID GSE287409.
