# Supplementary material for: A bioinformatics screen identifies TCF19 as an aggressiveness‐sustaining gene in prostate cancer
Source: Mol Oncol. 2025 Sep 15;19(12):3634–50. doi: 10.1002/1878-0261.70118 (PMC12688178; doi:10.1002/1878-0261.70118)
Supplement: Supplementary file 1 — Fig. S1. Related to Fig. 1. Fig. S2. Related to Fig. 2. Fig. S3. Related to Fig. 2. Fig. S4. Related to Fig. 2. Fig. S5. Related to Fig. 3. Fig. S6. Related to Fig. 4. Fig. S7. Related to Fig. 4. Fig. S8. Related to Fig. 5. Fig. S9. Related to Fig. 6. Fig. S10. Related to Fig. 6. Fig. S11. Related to Fig. 7. [file MOL2-19-3634-s003.pdf]

Supplementary Figure 1

Dataset	Cohort size		
	Normal	*Primary Tumor*	*Metastasis*
**Grasso**	12	49	27
**Lapointe**	9	13	4
**Taylor**	29	131	19
**Tomlins**	23	32	20
**Varambally**	6	7	6

Dataset	DFS.Status = 0	DFS.Status = 1	Missing data - with respect to PT	DFS.TIME min	DFS.TIME max	DFS.TIME mean
**Fraser**	57	16	0	1.64	154.45	74.19
**Glinsky**	42	37	0	1.4	105.7	51.54
**Taylor**	104	27	0	1.38	149.19	48.19
**TCGA**	400	91	6	0.76	165.05	32.14

Supplementary Figure 1. *Related to Fig 1*. Information related to the cohort size and Disease-Free Survival (DFS) of the datasets (PMID: 22722839) (PMID: 14711987) (PMID: 20579941) (PMID: 17173048) (PMID: 16286247) (PMID: 28068672) (PMID: 15067324) (https://gdac.broadinstitute.org/) used for the computational screening.

Supplementary Figure 2

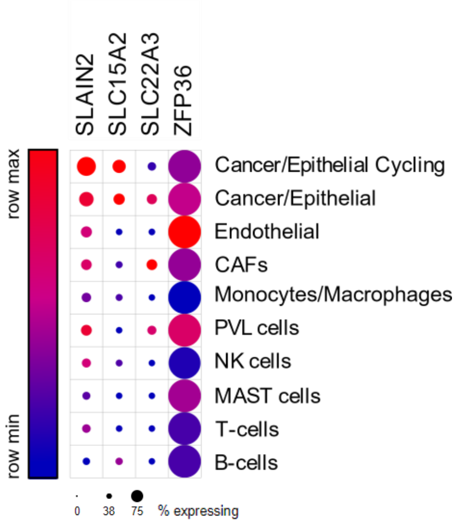

Supplementary Figure 2. *Related to Fig 2.* The relative expression of each gene in the indicated cell type was retrieved from the single cell data from a prostate cancer study (PMID: 33971952). The size of the dot represents the % of expressing cells.

Supplementary Figure 3

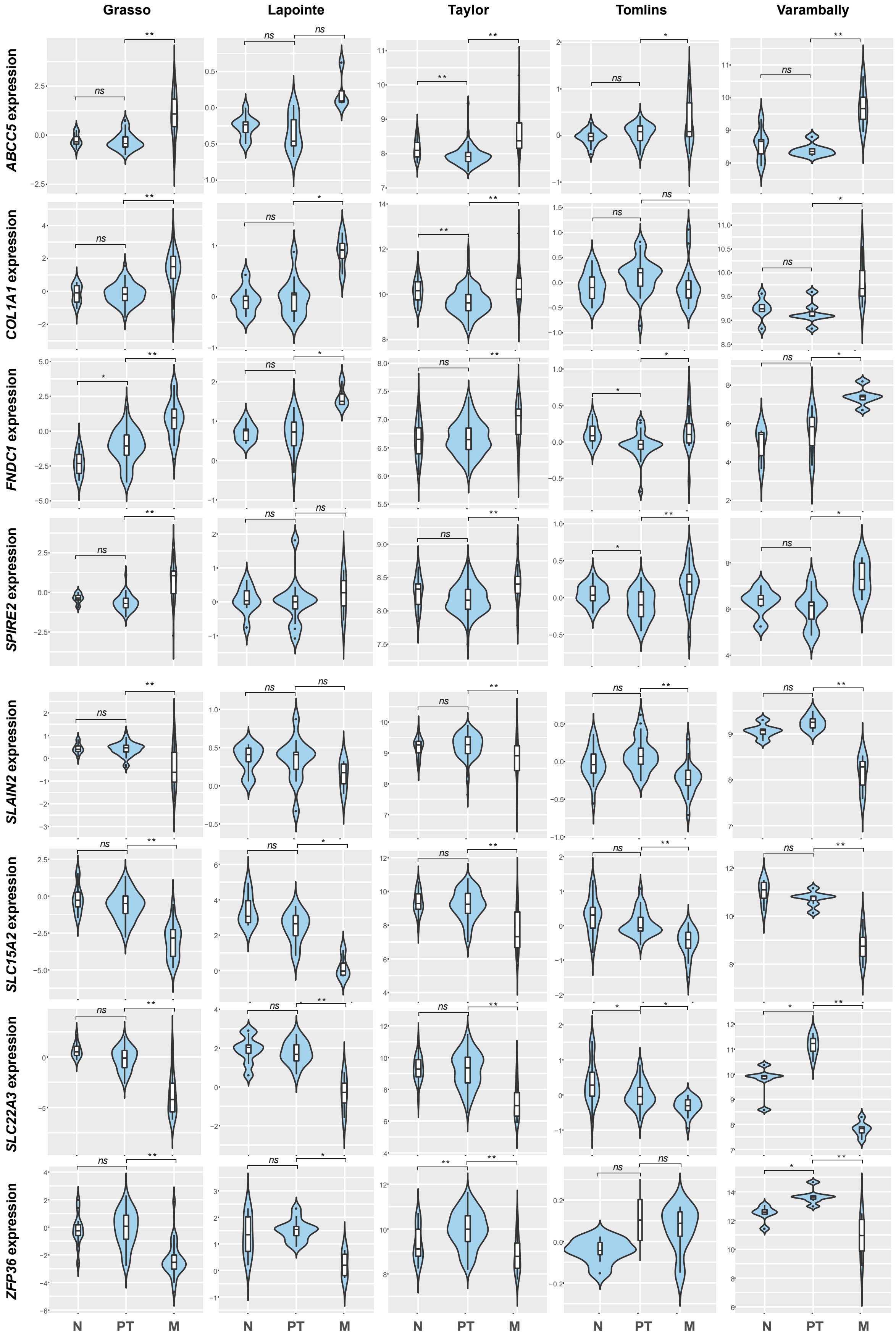

Supplementary Figure 3. *Related to Fig 2.* Violin plots depicting the expression of the indicated gene among non-tumoral (N), primary tumor (PT) and metastatic (M) prostate cancer specimens in the indicated datasets. The y-axis represents the Log_2-normalized gene expression (fluorescence intensity values for microarray data or, sequencing reads values obtained after gene quantification with RSEM and normalization using Upper Quartile in case of RNA-seq). p value derives from the limma differential expression between the indicated groups in the computational analysis *(p,p-value. ns p≥0.05, * p<0.05, ** p<0.005, *** p<0.0005).*

Supplementary Figure 4

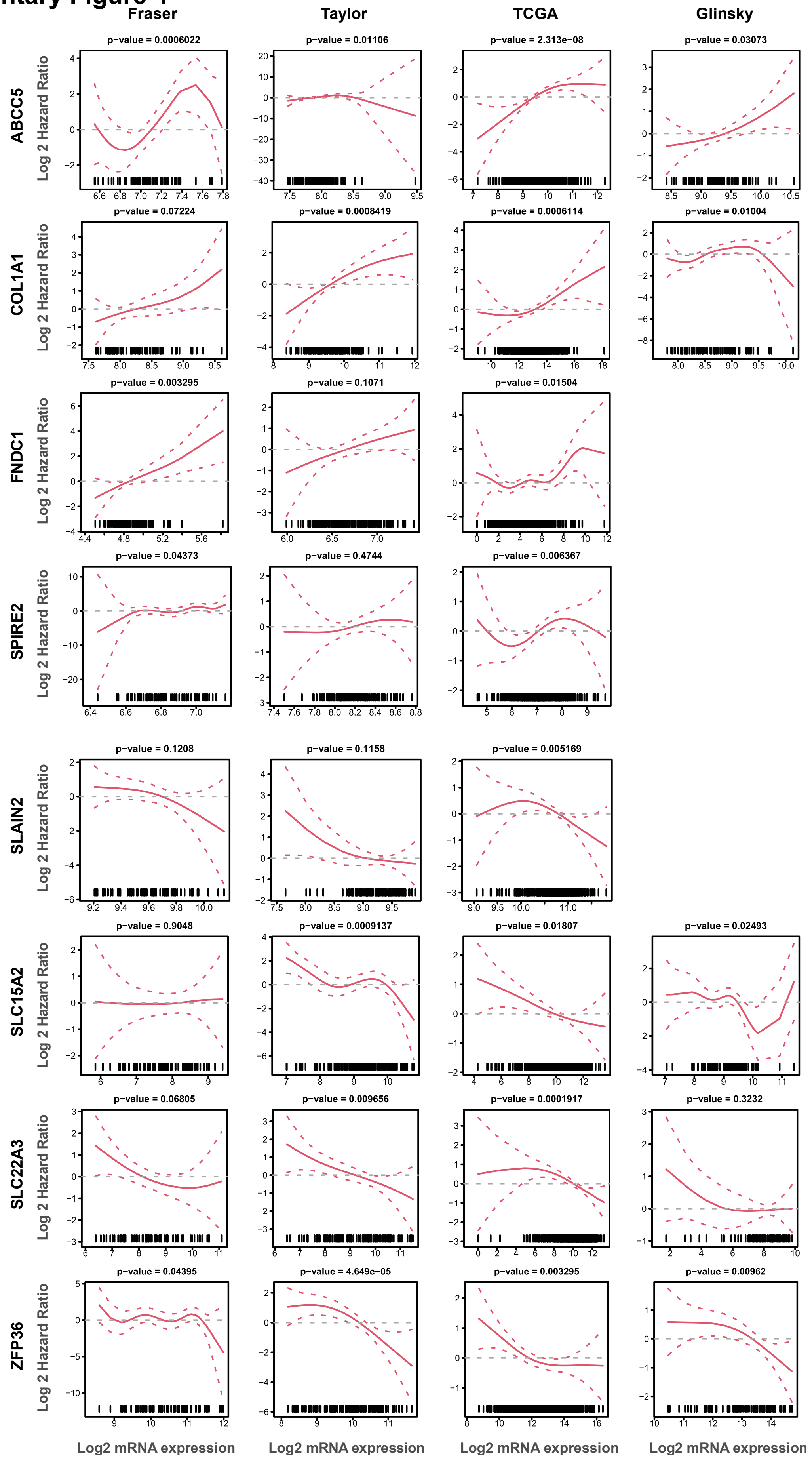

Supplementary Figure 4. *Related to Fig 2.* Smooth Hazard ratio curves. x-axis represents the gene expression level and y-axis the Log Hazard ratio. The p-value indicates the significance of the association between the gene and the outcome calculated via a likelihood ratio test.

Supplementary Figure 5

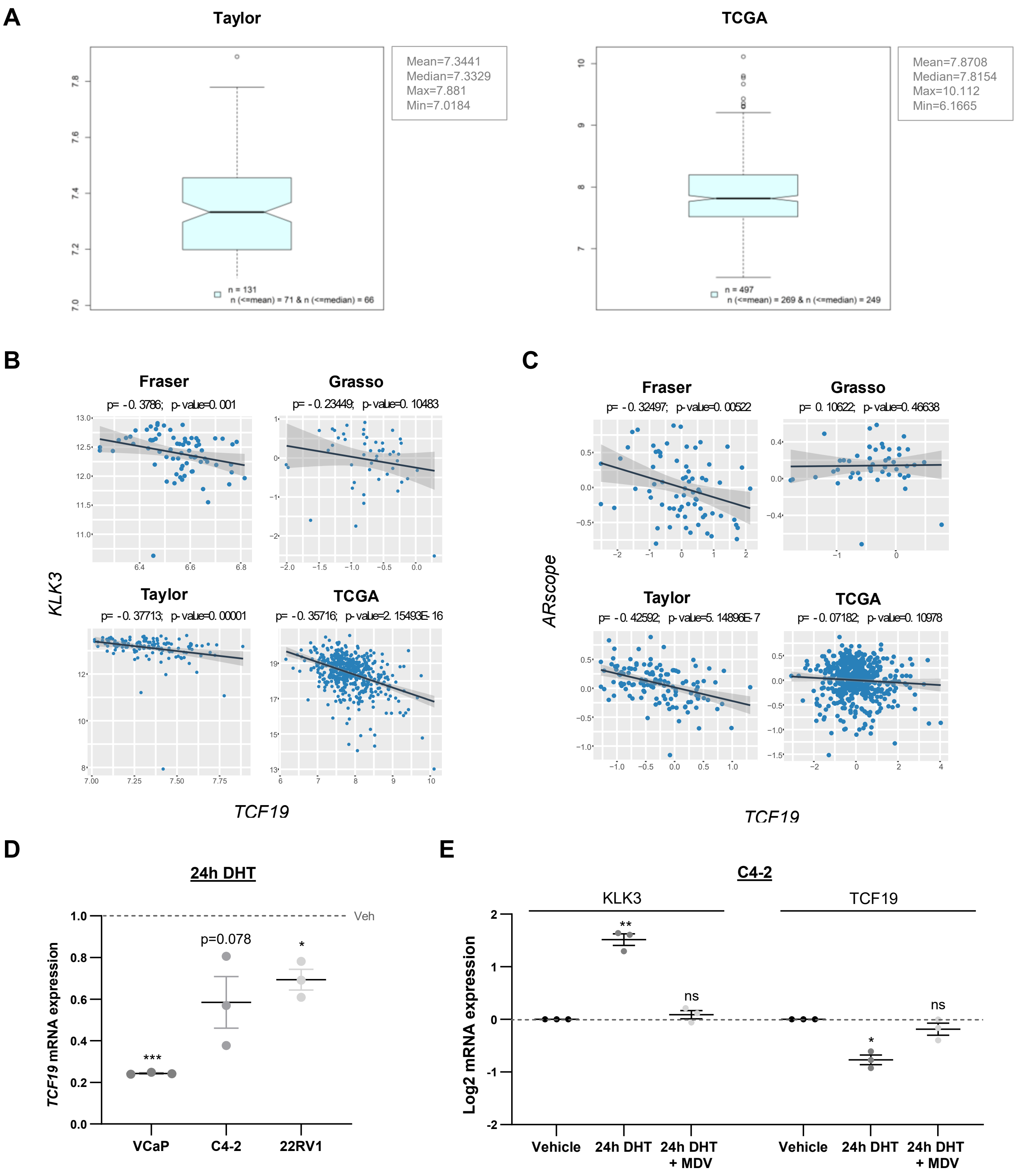

Supplementary Figure 5. *Related to Fig 3.* **A)** Box plots showing the distribution of patients based on their *TCF19* expression levels. The cohort size and the mean, median, max and min values of *TCF19* expression in each indicated dataset are shown. **B and C)** Plotted values correspond to the Log_2-normalized gene expression values of the indicated genes or AR signature from (PMID: 17010675) (in X and Y-axis) in the primary tumor specimens from each patient in the indicated dataset. Black line represents linear regression, grey area indicates the limits of the confidence intervals and p and p-value indicate Spearman's correlation coefficient and statistical significance respectively. **D)** Analysis of *TCF19* expression by qRT-PCR in the indicated cell line treated for 24h with AR agonist (dihydrotestosterone, DHT, 10 nM). Data were normalized to *GAPDH* expression and untreated (Veh; vehicle) condition. The dotted line represents the normalized value of the vehicle data. A one sample *t*-test was performed. Error bars indicate SEM. *n* = 3 independent experiments. **E)** Analysis of *KLK3* and *TCF19* expression by qRT-PCR in the AR-dependent C4-2 cells treated for 24h with AR agonist (dihydrotestosterone, DHT, 10 nM) alone or in combination with the AR antagonist (Enzalutamide, MDV 3100, 10μM). Data were normalized to *GAPDH* expression and untreated (vehicle) condition. The dotted line represents the normalized value of the vehicle data. A one sample *t*-test was performed. Error bars indicate SEM. *n* = 3 independent experiments. *p, p-value. ns p≥0.05, * p<0.05, ** p<0.01, *** p<0.001.*

Supplementary Figure 6

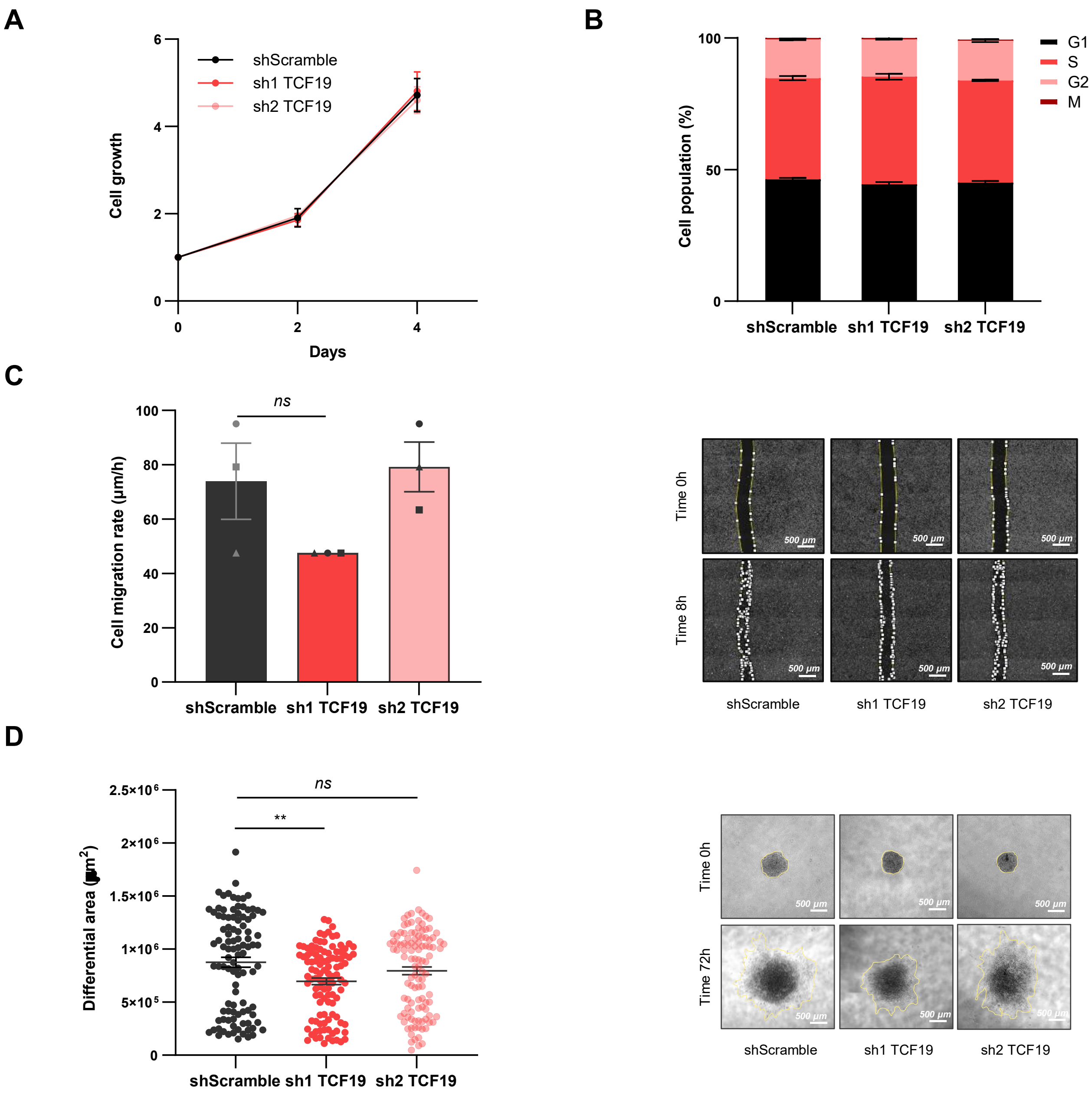

<u>**Supplementary Figure 6.**</u> *Related to Fig 4.* **A)** Analysis of cell proliferation of PC3 cells upon *TCF19* silencing. The cell number at each time point relative to time 0 are represented. A multiple paired *t*-test was applied for statistical analysis. Error bars represent SEM. *n*=3 independent experiments. **B)** Cell cycle distribution of shScramble or shTCF19 transduced PC3 cells. A multiple paired *t*-test was applied for statistical analysis. Error bars represent SEM. *n*=3 independent experiments. **C)** Analysis of cell migration rate of PC3 cells transduced with the indicated shRNA. The different biological replicates are indicated with unique dot shapes (left panels). A two-tailed paired Student's *t*-test was applied for statistical analysis. Error bars represent SEM. *n*=3 independent experiments. Representative images of the scratch at initial and final timepoint are shown (right panels). The area defined by the white dots and yellow lines was used for the measurements. Scale bar: 500 µm. **D)** Analysis of invasive growth of PC3 cells transduced with the indicated shRNA. Cell spheroids were embedded in collagen and measured at 0 and 72 hours (h). The differential area between final and initial timepoint was measured (left panel). A two-tailed unpaired Student's *t*-test was applied for statistical analysis. Scale bar: 500 µm. Error bars represent SEM. *n*=6, independent analysis. Representative images of the spheroids at final timepoint are shown (right panel). The yellow line represents the measured area. *p, p-value. ns p≥0.05, * p<0.05, ** p<0.01, *** p<0.001.*

Supplementary Figure 7

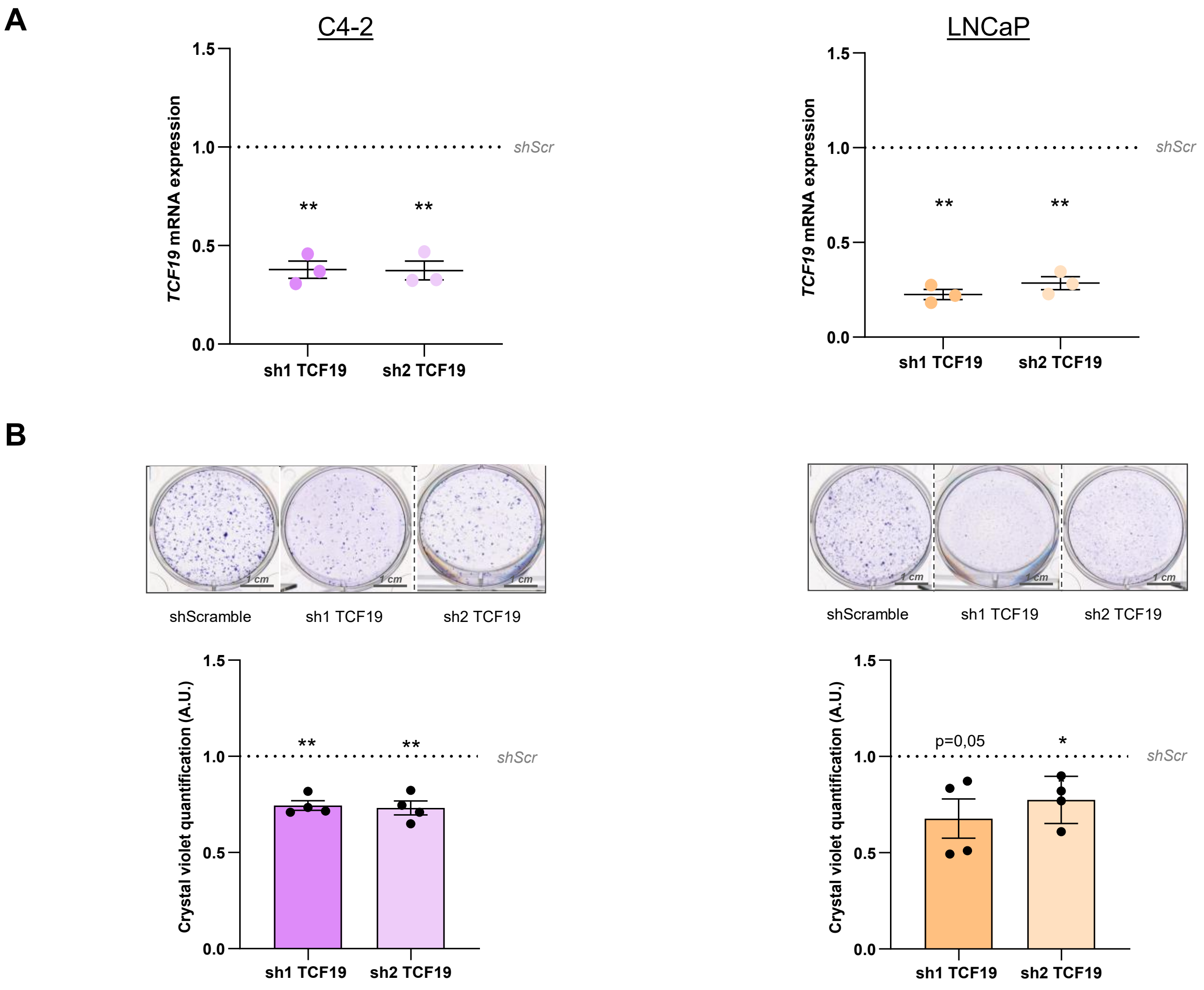

Supplementary Figure 7. *Related to Fig 4*. **A)** Analysis of *TCF19* gene expression by qRT-PCR in the indicated cell lines upon silencing of *TCF19* by shRNA transduction. Data were normalized to *GAPDH* expression and shScramble (shScr) condition. The dotted line represents the normalized value of the shScramble data. A one sample *t*-test was performed for statistical analysis. Error bars represent SEM. *n*=3 independent experiments. **B)** Analysis of foci formation upon *TCF19* depletion. Crystal violet solubilized intensity normalized to shScramble condition is shown (lower panels). A one sample *t*-test was performed for statistical analysis. Error bars represent SEM. *n*=4 independent experiments. Representative images are shown (Upper panels). Scale bar: 1 cm. p, p-value. ns p≥0.05, * p<0.05, ** p<0.01.

Supplementary Figure 8

A

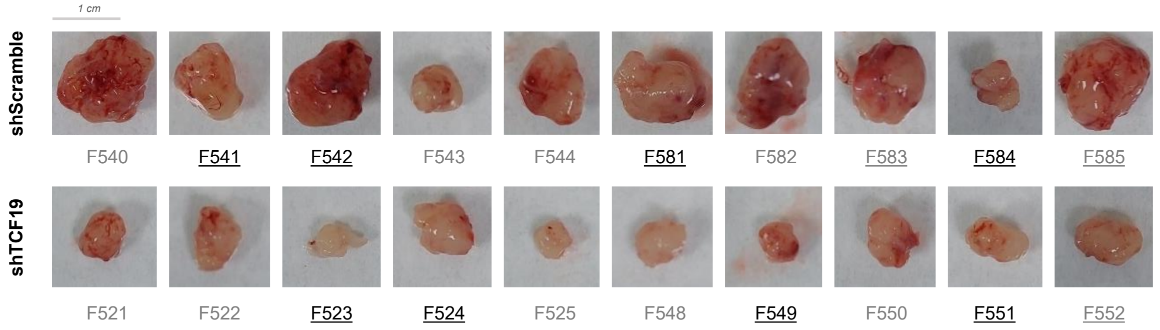

B

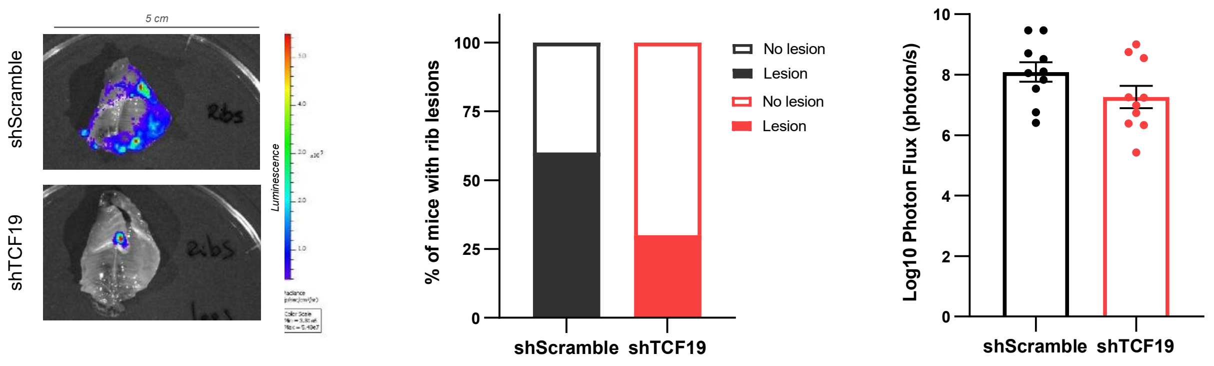

Supplementary Figure 8. *Related to Fig 5.* **A)** Images of the tumours collected at the end of the orthotopic xenotransplant assay. Scale bar: 1 cm. The tumors characterized in Figure 6 are indicated underline (qPCR) and grey (IHC/IF). **B)** Evaluation of metastatic lesions in the ribs by orthotopic xenotransplant assay. Representative images (left panel). Scale bar: 5 cm. The *ex vivo* incidence of rib lesions (middle panel). Luciferase signal above day 0 was considered metastasis-positive. The Log_{10} photon flux signal of the ribs are represented (right panel). A two-sided Fisher's exact test was performed for statistical analysis. Error bars represent SEM. *p, p-value. ns p≥0.05, * p<0.05, ** p<0.01, *** p<0.001.*

Supplementary Figure 9

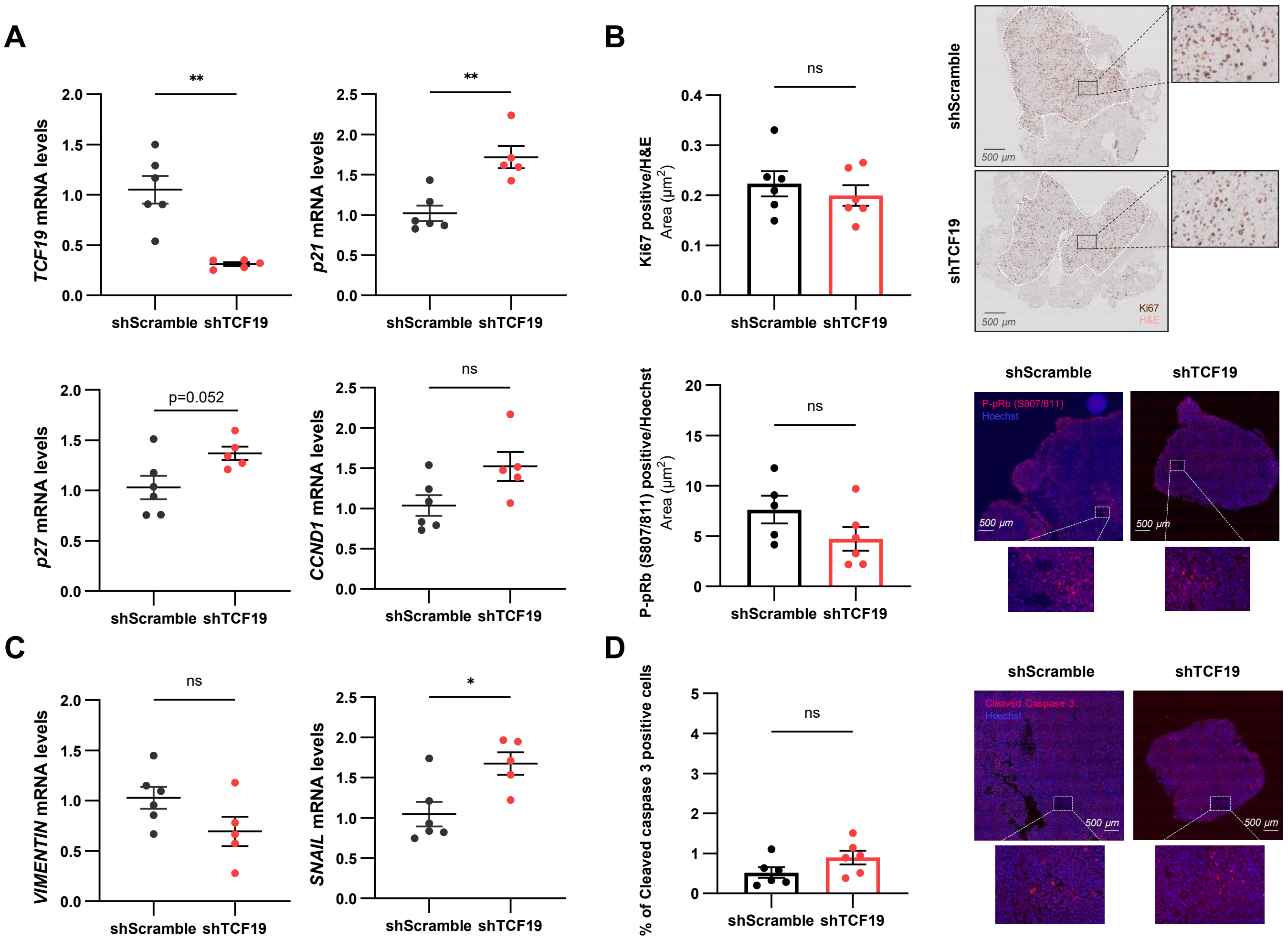

<u>Supplementary Figure 9.</u> *Related to Fig 6.* **A)** Gene expression analysis of shScramble and shTCF19 tumors from the *in vivo* orthotopic experiment. The tumors selected for the qRT-PCR analysis are indicated in Supplementary Fig. 7. Data were normalized to *GAPDH* expression and shScramble condition. A Mann-Whitney *U*-test was performed for statistical analysis. Error bars represent SEM. *n* = 6 tumors/group. **B)** Analysis of Ki67 and P-pRb (S807/811) in shScramble and shTCF19 tumors from the *in vivo* orthotopic experiment. The tumors selected for the IHC/IF analysis are indicated in Supplementary Fig. 7. A Mann-Whitney *U*-test was performed for statistical analysis (left panels). Error bars represent SEM. *n* = 6 tumors/group. Representative images are shown (right panels). **C)** Gene expression analysis of shScramble and shTCF19 tumors from the *in vivo* orthotopic experiment. The tumors selected for the qRT-PCR analysis are indicated in Supplementary Fig. 7. Data were normalized to *GAPDH* expression and shScramble condition. A Mann-Whitney *U*-test was performed for statistical analysis. Error bars represent SEM. *n* = 6 tumors/group. **D)** Analysis of Cleaved Caspase 3 in shScramble and shTCF19 tumors from the *in vivo* orthotopic experiment. The tumors selected for the IF analysis are indicated in Supplementary Fig. 7. A Mann-Whitney *U*-test was performed for statistical analysis (left panels). Error bars represent SEM. *n* = 6 tumors/group. Representative images are shown (right panels). Scale bar: 500 µm. *ns p≥0.05, * p<0.05, ** p<0.01.*

Supplementary Figure 10

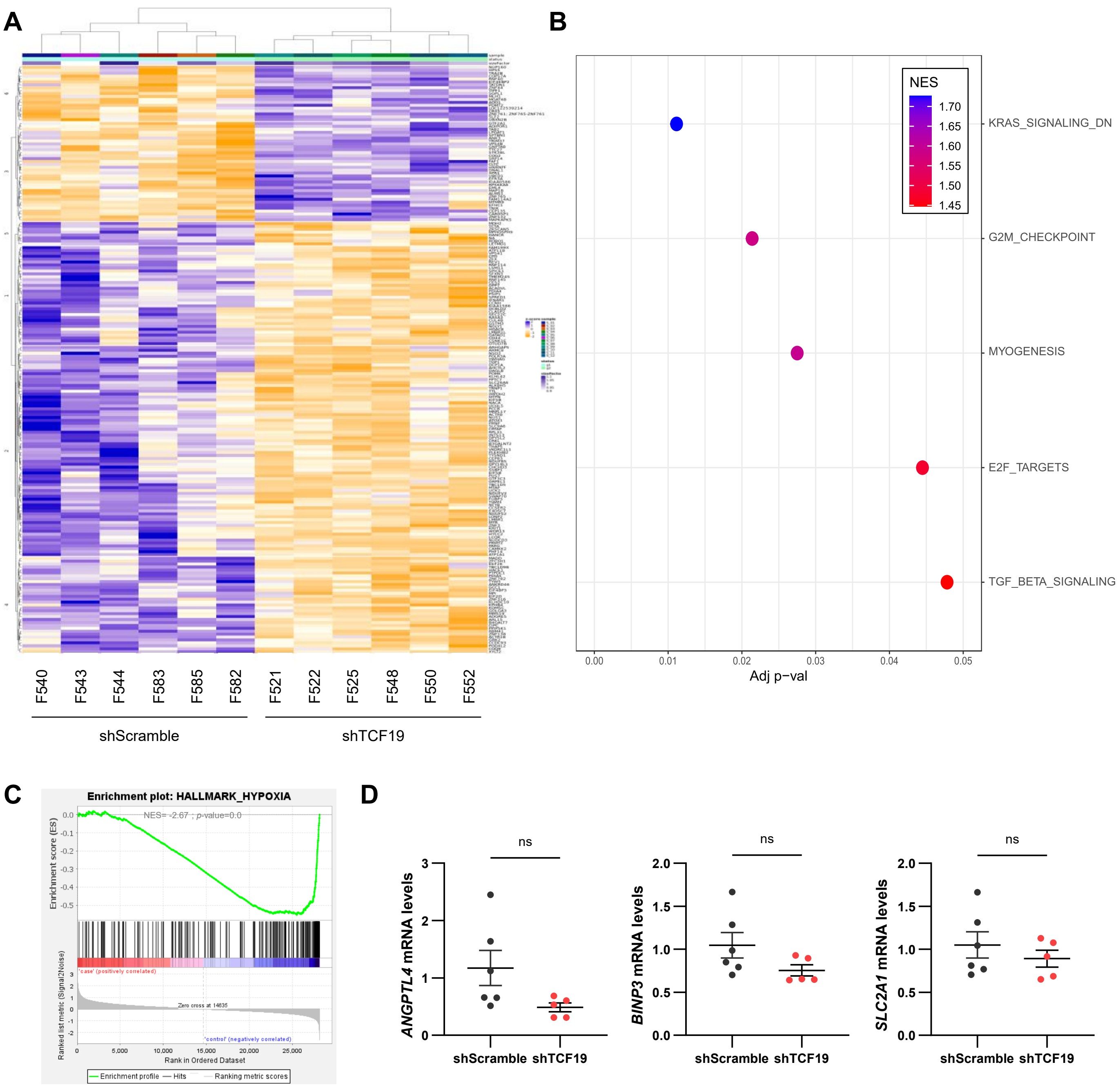

<u>Supplementary Figure 10.</u> *Related to Fig 6*. **A)** The Z-scored heatmap representing the Top200 significant changes ($p<0.05$) upon *TCF19* depletion in the RNA-seq analysis. **B and C)** A GSEA analysis was performed to identify pathways enriched upon *TCF19* depletion. The Top 5 pathways phenotypically enriched in the shTCF19 condition at the GSEA analysis are shown (B). The GSEA profile of a selected pathway from Fig. 6B is shown (C). **D)** Gene expression analysis of shScramble and shTCF19 tumors from the *in vivo* orthotopic experiment. The tumors selected for the qRT-PCR analysis are indicated in Supplementary Fig. 7. Data were normalized to *RPLP0* expression and shScramble condition. A Mann-Whitney *U*-test was performed for statistical analysis. Error bars represent SEM. *n* = 6 tumors/group. *p, p-value. ns p≥0.05.*

Supplementary Figure 11

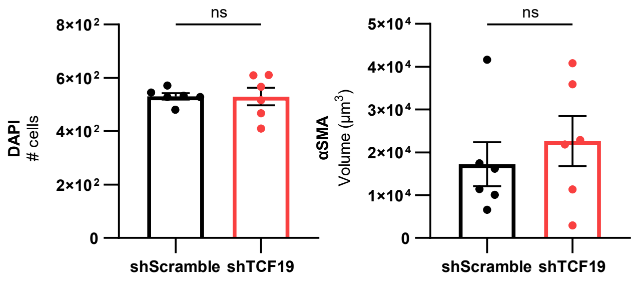

<u>Supplementary Figure 11.</u> *Related to Fig 7*. Analysis of total αSMA and DAPI in shScramble and shTCF19 tumors from the *in vivo* orthotopic experiment. The tumors selected for the IHC/IF analysis are indicated in Supplementary Fig. 7. A Nested *t*-test was performed for statistical analysis. Error bars represent SEM. n = 6 tumors/group. *p, p-value. ns* $p \geq 0.05$.
